# Adverse drug reaction signals mining comparison of amiodarone and dronedarone: a pharmacovigilance study based on FAERS

**DOI:** 10.3389/fphar.2024.1438292

**Published:** 2024-10-22

**Authors:** Ye Xu, Bin Zhao, Liqun He

**Affiliations:** ^1^ Department of Cardiology, Intervention Cardiology Center, Wuhan No. 1 Hospital, Wuhan, China; ^2^ Xiamen Health and Medical Big Data Center (Xiamen Medical Research Institute), Xiamen, Fujian, China

**Keywords:** amiodarone, dronedarone, data mining, FAERS, pharmacovigilance, adverse drug reaction

## Abstract

**Background:**

Amiodarone and dronedarone are both class III antiarrhythmic medications used to treat arrhythmias. The objective of this study was to enhance the current understanding of adverse drug reaction (ADR) associated with amiodarone and dronedarone by employing data mining methods on the U.S. Food and Drug Administration Adverse Event Reporting System (FAERS), and providing a reference for safe and reasonable clinical use.

**Methods:**

The ADR records were selected by searching the FAERS database from 2011 Q3 to 2023 Q3. The disproportionality analysis algorithms, including Reporting Odds Ratio (ROR), Proportional Reporting Ratio (PRR), Bayesian Confidence Propagation Neural Network (BCPNN), and Empirical Bayesian Geometric Mean (EBGM), were used to detect signals of amiodarone-related and dronedarone-related ADRs. The ADR profiles of amiodarone and dronedarone categorized by organ toxicity were compared through the Z-test and the Fisher exact test.

**Results:**

9,295 reports specifically mentioned the use of amiodarone and 2,485 reports mentioned the use of dronedarone among 9,972,109 reports, with the majority of ADRs occurring in males over 60 years old. The United States was responsible for the highest proportion of reported ADRs. Significant system organ classes (SOC) for both included Cardiac disorders, Respiratory, thoracic and mediastinal disorders, and Investigations, etc. At the preferred terms (PTs) level, the more frequent ADR signals for amiodarone were drug interaction (n = 856), hyperthyroidism (n = 758), and dyspnoea (n = 607), while dronedarone were atrial fibrillation (n = 371), dyspnoea (n = 204), and blood creatinine increased (n = 123). Notably, unexpected ADRs, including electrocardiogram T wave alternans (n = 16; EBGM05 = 231.27), accessory cardiac pathway (n = 11; EBGM05 = 140), thyroiditis (n = 178; EBGM05 = 125.91) for amiodarone, and cardiac ablation (n = 11; EBGM05 = 31.86), cardioversion (n = 7; EBGM05 = 22.69), and dysphagia (n = 47; EBGM05 = 3.6) for dronedarone, were uncovered in the instructions. The analysis also revealed significant differences in the ADR profiles of amiodarone and dronedarone, with dronedarone showing higher proportions of cardiac toxicity but lower thyroid toxicity compared to amiodarone.

**Conclusion:**

These findings underscore the significance of vigilantly monitoring and comprehending the potential risks linked to the use of amiodarone and dronedarone. New ADRs discovered and clear ADR profiles of amiodarone and dronedarone enhance a thorough understanding of these drugs, which is essential for clinicians to ensure safe use of amiodarone and dronedarone.

## 1 Introduction

Amiodarone, as a class III antiarrhythmic medication blocking multiple ion channels, has been widely used to treat both supraventricular and ventricular arrhythmias (Singh and Vaughan Williams, 1970). However, its extensive use over the years has revealed plenty of adverse effects, ranging from thyroid toxicity to pulmonary toxicity and liver toxicity. These drawbacks have prompted the exploration of alternative treatments with comparable efficacy but fewer adverse reactions. Dronedarone, a structurally similar compound designed to address the limitations of amiodarone by targeting similar cardiac ion channels, aims to provide effective rhythm control while minimizing the risk of systemic toxicities associated with its predecessor (Hohnloser et al., 2009; Torp-Pedersen et al., 2011). Nonetheless, careful monitoring of adverse drug reactions (ADRs) and the acquisition of real-world data are crucial for establishing clinically valid drug reference standards, and ensuring both drugs are used optimally to maximize patient benefits and minimize risks.

The U.S. Food and Drug Administration Adverse Event Reporting System (FAERS) is a publicly accessible database specifically designed to assist the FDA in post-market safety monitoring of drugs and therapeutic biologic products (Wang et al., 2024; Zhang et al., 2024). This database allows for the quantitative assessment of reports through signal detection, where a “signal” indicates a drug-related adverse event and is then analyzed to determine if it is an ADR or not. Researchers utilize a variety of particle-based analysis algorithms, such as the Reporting Odds Ratio (ROR) (Rothman et al., 2004), Proportional Reporting Ratio (PRR) (Evans et al., 2001), Bayesian Confidence Propagation Neural Network (BCPNN) (Bate et al., 1998), and Empirical Bayesian Geometric Mean (EBGM) (Dumouchel, 1999), to evaluate AE risks and detect the signal strength of adverse events associated with medical products. This enables researchers to analyze a wide range of drugs and diseases, identifying potential ADRs of particular concern and thus providing guidance for clinical medication and treatment strategies (Zhao et al., 2024; Du et al., 2024a; Du et al., 2024b).

In this study, we analyzed amiodarone-related and dronedarone-related ADRs reported from Q3 2011 to Q3 2023, utilizing data mining from the FAERS database. We employed the ROR and PRR algorithms to assess the associations between the drugs and adverse events, thereby filtering out ADRs. Subsequently, we constructed a joint probability model linking the drugs to the ADRs identified through ROR and PRR, using the BCPNN algorithm. Lastly, we applied the EBGM algorithm to convert these risk associations into corresponding risk indices, identifying significant high-risk combinations of drugs and ADRs as recognized by the aforementioned algorithms. Additionally, we conducted a comparative analysis of the ADRs associated with both drugs, uncovering new ADR signals. We believe this research will enhance the rational and safe clinical use of amiodarone and dronedarone.

## 2 Materials and methods

### 2.1 Data source and collection

The FAERS is a publicly accessible database designed to facilitate post-marketing surveillance of drugs and therapeutic biologics, capturing all relevant adverse event and medication error information reported by consumers and healthcare professionals. Covering the period from Q3 2011 to Q3 2023, data pertaining to amiodarone and dronedarone ADRs was extracted and preprocessed using MySQL. This clean and standardized data was then meticulously selected and mapped to MedDRA 23.0 to eliminate duplicate case records and precisely identify the adverse events. Significant adverse events were further categorized into preferred terms (PTs) and system organ classes (SOCs) according to the structured hierarchy of MedDRA terminology (Mascolo et al., 2021). Additionally, comprehensive data on clinical characteristics such as gender, age, and reporting countries were systematically compiled for analysis. Severe outcomes, including life-threatening conditions, hospitalization, disability, and death, were also examined. The search terms for amiodarone were “amiodarone” and “amiodarone HYDROCHLORIDE”; for dronedarone, “dronedarone” and “MULTAQ”. ADRs were systematically classified and detailed according to PTs and SOCs in the International MedDRA, version 25.1. This thorough approach ensures an in-depth evaluation of the safety profiles of these medications.

### 2.2 Statistical analysis

Disproportionality analysis serves as a fundamental technique in pharmacovigilance to identify spontaneous adverse event signals (Zink et al., 2013). In this study, we employed both Frequentist and Bayesian methods to investigate potential correlations between amiodarone or dronedarone and their respective ADRs, using established statistical tools including the ROR, PRR, BCPNN, and EBGM. We limited our analysis to adverse event signals with a minimum of three records. These signals were required to simultaneously meet all four algorithmic criteria, notably a lower limit of the 95% confidence interval (CI) greater than 1.0. To reduce the likelihood of false positives associated with infrequent events with minimal case numbers, we applied a statistical shrinkage technique (Norén et al., 2013). At the PTs level, the number of ADRs and ADR types were then categorized by organ toxicity, and the Z-test and the Fisher exact test for proportions were used to compare the proportions of ADRs and ADR types between amiodarone and dronedarone. All data processing and statistical analyses were performed using R software, version 4.3.3.

Before calculating the ROR and PRR, it is essential to determine the values of a, b, c, and d. In this context, “a” represents the number of individuals who experienced the target adverse event post-drug exposure, “b” indicates the number of individuals who experienced non-target adverse events post-drug exposure, “c” refers to the number of individuals who experienced the target adverse event post non-drug exposure, and “d” represents the number of individuals who experienced non-target adverse events post non-drug exposure. Consequently, the total number of cases, denoted as “N”, is computed as N = a + b + c + d. This calculation is illustrated in Table 1. The precise formulas for the four algorithms are outlined subsequently:

(i) ROR algorithm

$$ROR=\left(ad\right)/\left(bc\right)$$

$$95\%CI=e^{In\left(ROR\right)\pm 1.96\sqrt{\frac{1}{a}+\frac{1}{b}+\frac{1}{c}+\frac{1}{d}}}$$

**TABLE 1:** Table matrix.

	Amiodarone/dronedarone	Non-amiodarone/dronedarone
Target AEs	a	c
Non-target AEs	b	d
N = a + b + c + d		

The criteria of positive safety signal detection: the lower limit of 95% *CI* > 1, *N* ≥ 3;

(ii) PRR algorithm

$$PRR=\left[a\;\left(c+d\right)\right]/\left[c\;\left(a+b\right)\right]$$

$$\chi 2=\frac{\left(a+b+c+d\right){\left(ad\;—\;bc\right)}^{2}}{\left(a+b\right)\left(c+d\right)\left(a+c\right)\left(b+d\right)}$$

The criteria of positive safety signal detection: PRR ≥ 2, χ2 ≥ 4, *N* ≥ 3;

(iii) BPCNN algorithm

$$IC=\log_{2}\frac{a\left(a+b+c+d\right)}{\left(a+b\right)\left(a+c\right)}$$

$$95\%CI=E\left(IC\right)\pm 2\times \sqrt{V\left(IC\right)}$$

The criteria of positive signals: IC025 > 0 (IC025 represents the lower bound of 95% CI);

(iv) EBGM algorithm

$$EBGM=\left(aN\right)/\left[\left(a+b\right)\left(a+c\right)\right]$$

$$95\%CI=e^{In\left(EBGM\right)\pm 1.96\sqrt{\frac{1}{a}+\frac{1}{b}+\frac{1}{c}+\frac{1}{d}}}$$

The criteria of positive safety signal detection: EBGM05 > 2 (EBGM05: the lower bound of 95% CI).

## 3 Results

### 3.1 ADR reports and clinical characteristics

From Q3 2011 to Q3 2023, a total of 9,972,109 reports were submitted to the FAERS database. Out of these, 9,295 reports specifically mentioned the use of amiodarone, and 2,485 reports mentioned the use of dronedarone. The clinical characteristics of events with amiodarone and dronedarone are detailed in Table 2. Among all ADRs, a higher percentage of male than female patients (54.23% vs. 34.18%) was reported with the use of amiodarone, while dronedarone showed the opposite trend and had a higher percentage of female than male (47.53% vs. 40.64%). The most significant percentage of reports of amiodarone (39.43%) and dronedarone (41.93%) both occurred in patients aged 60–79 years. The United States is the main drug reporting country for both amiodarone (44.29%) and dronedarone (73.20%), but it is more concentrated for dronedarone. As for severe outcomes, hospitalization was the most frequently reported for amiodarone and dronedarone (49.14% vs. 23.30%), and the incidence of disability was also the lowest for both (3.34% vs. 2.05%).

**TABLE 2:** The characteristics of reports associated with amiodarone or dronedarone from the FAERS database (2011 Q3 to 2023 Q3).

	Amiodarone (n, %)	Dronedarone (n, %)
Total	9,295 (100.00)	2,485 (100.00)
Gender		
Male	5,052 (54.35)	1,010 (40.64)
Female	3,192 (34.34)	1,181 (47.53)
Unknown	1,051 (11.31)	294 (11.83)
Age		
˂19	182 (1.96)	3 (0.12)
20–39	218 (2.35)	14 (0.56)
40–59	992 (10.67)	174 (7.00)
60–79	3,745 (40.29)	1,042 (41.93)
≥80	2,123 (22.84)	446 (17.95)
Unknown	2,035 (21.89)	806 (32.43)
Reported countries (the top ranked)		
US (United States)	4,117 (44.29)	1,819 (73.20)
FR (France)	1,337 (14.38)	193 (7.77)
IT (Italy)	717 (7.71)	63 (2.54)
DE (Germany)	582 (6.26)	58 (2.33)
UK (United Kingdom)	512 (5.51)	58 (2.33)
CA (Canada)	318 (3.42)	25 (1.11)
Others	1,712 (18.42)	292 (10.82)
Serious outcomes		
Hospitalization	4,568 (49.14)	579 (23.30)
Life-threatening	1,112 (11.96)	66 (2.66)
Death	1,070 (11.51)	124 (4.99)
Disability	310 (3.34)	51 (2.05)

### 3.2 SOC level disproportionality analysis

The signal strengths of reports of amiodarone and dronedarone at the SOC level are outlined in Tables 3, 4 separately. Analyzing the data, amiodarone-associated ADRs affected 27 organ systems, underscoring that ADRs related to amiodarone constitute a relatively common phenomenon. While dronedarone-associated ADRs affected 15 organ systems, these also involved a wide range of aspects. Predominantly, the largest numbers of ADRs were observed in Cardiac disorders in both amiodarone and dronedarone (n = 3,880, EBGM05 = 8.04 vs. n = 825, EBGM05 = 11.3), compliant with drug pharmacology and our expectations. Other large numbers of ADRs for both were observed in Respiratory, thoracic, and mediastinal disorders (n = 2,876, EBGM05 = 5.93 vs. n = 312, EBGM05 = 4.35), and Investigations (n = 1,795, EBGM05 = 8.31 vs. n = 550, EBGM05 = 7.67). Moreover, it was discovered that Surgical and medical procedures (n = 27, EBGM05 = 9.82), and Infections and infestations (n = 8, EBGM05 = 56.4) at the SOC level represent new ADRs that are not labeled in the instructions for amiodarone, and Surgical and medical procedures (n = 35, EBGM05 = 11.7) represent new ADRs for dronedarone. The 95% CI for the ROR only shows the lower limit of the 95% two-sided CI of the ROR.

**TABLE 3:** Signal strength of AEs of amiodarone at the system organ class (SOC) level in FDA adverse event reporting system (FAERS) source.

SOC code	SOC	Case reports	ROR (lower_95% CI)	PRR (χ2)	IC (IC025)	EBGM (EBGM05)
10007541	Cardiac disorders	3,880	13.77 (13.22)	8.44 (26579.13)	3.07 (2.94)	8.38 (8.04)
10038738	Respiratory, thoracic and mediastinal disorders	2,876	8.58 (8.21)	6.23 (13220.33)	2.63 (2.49)	6.2 (5.93)
10027433	Metabolism and nutrition disorders	2,008	23.3 (22.17)	18.48 (33037.5)	4.18 (4.02)	18.19 (17.3)
10022891	Investigations	1,795	10.69 (10.15)	8.82 (12618.64)	3.13 (2.96)	8.75 (8.31)
10018065	General disorders and administration site conditions	1,713	7.32 (6.95)	6.16 (7583.21)	2.62 (2.44)	6.13 (5.81)
10019805	Hepatobiliary disorders	801	6.82 (6.34)	6.32 (3612.67)	2.65 (2.41)	6.29 (5.84)
10047065	Vascular disorders	744	6.28 (5.83)	5.86 (3025.05)	2.54 (2.29)	5.84 (5.41)
10029205	Nervous system disorders	679	4.78 (4.42)	4.5 (1872.69)	2.17 (1.9)	4.49 (4.15)
10022117	Injury, poisoning and procedural complications	619	6.8 (6.26)	6.41 (2839.24)	2.67 (2.4)	6.38 (5.88)
10021428	Immune system disorders	572	20.68 (18.99)	19.47 (9873.89)	4.26 (3.97)	19.14 (17.57)
10014698	Endocrine disorders	325	46.6 (41.63)	45.01 (13431.65)	5.43 (5.06)	43.23 (38.62)
10038359	Renal and urinary disorders	230	2.8 (2.46)	2.76 (259.5)	1.46 (1.02)	2.75 (2.42)
10005329	Blood and lymphatic system disorders	227	6.9 (6.05)	6.76 (1110.84)	2.75 (2.31)	6.72 (5.89)
10015919	Eye disorders	222	26.73 (23.36)	26.11 (5238.56)	4.67 (4.22)	25.51 (22.3)
10040785	Skin and subcutaneous tissue disorders	214	6.27 (5.48)	6.15 (921.5)	2.61 (2.16)	6.12 (5.34)
10037175	Psychiatric disorders	146	13.38 (11.35)	13.19 (1626.81)	3.71 (3.16)	13.04 (11.06)
10028395	Musculoskeletal and connective tissue disorders	136	7.36 (6.21)	7.27 (732.15)	2.85 (2.29)	7.23 (6.1)
10017947	Gastrointestinal disorders	128	5.46 (4.59)	5.4 (457.87)	2.43 (1.85)	5.38 (4.52)
10038604	Reproductive system and breast disorders	29	20.27 (14.03)	20.21 (519.82)	4.31 (3.13)	19.85 (13.74)
10042613	Surgical and medical procedures	27	14.58 (9.97)	14.54 (336.02)	3.84 (2.63)	14.36 (9.82)
10036585	Pregnancy, puerperium and perinatal conditions	26	9.07 (6.16)	9.05 (184.55)	3.17 (1.93)	8.98 (6.1)
10010331	Congenital, familial and genetic disorders	22	32.03 (20.95)	31.95 (640.59)	4.96 (3.61)	31.06 (20.31)
10041244	Social circumstances	13	15.1 (8.73)	15.08 (168.55)	3.9 (2.21)	14.88 (8.61)
10013993	Ear and labyrinth disorders	12	14.63 (8.28)	14.62 (150.18)	3.85 (2.11)	14.43 (8.16)
10077536	Product issues	11	262.32 (135.64)	262.01 (2298.32)	7.72 (5.71)	210.74 (108.97)
10021881	Infections and infestations	8	132.03 (63.33)	131.92 (925.52)	6.88 (4.68)	117.57 (56.4)
10029104	Neoplasms benign, malignant and unspecified (incl cysts and polyps)	8	17.66 (8.78)	17.64 (123.57)	4.12 (2.05)	17.37 (8.64)

**TABLE 4:** Signal strength of AEs of dronedarone at the system organ class (SOC) level in FDA adverse event reporting system (FAERS) source.

SOC code	SOC	Case reports	ROR (lower_95% CI)	PRR (χ2)	IC (IC025)	EBGM (EBGM05)
10007541	Cardiac disorders	825	17.94 (16.5)	12.31 (8790.81)	3.62 (3.35)	12.28 (11.3)
10022891	Investigations	550	10.56 (9.6)	8.44 (3700.33)	3.08 (2.76)	8.43 (7.67)
10038738	Respiratory, thoracic and mediastinal disorders	312	5.47 (4.85)	4.91 (994.76)	2.29 (1.9)	4.9 (4.35)
10018065	General disorders and administration site conditions	85	4.84 (3.9)	4.71 (250.06)	2.24 (1.52)	4.71 (3.79)
10017947	Gastrointestinal disorders	75	4.24 (3.37)	4.14 (179.95)	2.05 (1.29)	4.14 (3.29)
10038359	Renal and urinary disorders	74	3.04 (2.41)	2.98 (98.09)	1.57 (0.81)	2.98 (2.36)
10019805	Hepatobiliary disorders	53	5.62 (4.28)	5.52 (196.61)	2.46 (1.57)	5.51 (4.2)
10042613	Surgical and medical procedures	35	16.63 (11.9)	16.41 (504.95)	4.03 (2.95)	16.35 (11.7)
10040785	Skin and subcutaneous tissue disorders	30	4.36 (3.04)	4.31 (76.56)	2.11 (0.95)	4.31 (3.01)
10021428	Immune system disorders	29	6.55 (4.54)	6.48 (134.59)	2.7 (1.52)	6.48 (4.49)
10027433	Metabolism and nutrition disorders	24	17.23 (11.52)	17.07 (361.93)	4.09 (2.81)	17.01 (11.37)
10029205	Nervous system disorders	23	7.73 (5.12)	7.66 (133.25)	2.94 (1.63)	7.66 (5.08)
10022117	Injury, poisoning and procedural complications	19	6.64 (4.23)	6.6 (90.26)	2.72 (1.3)	6.59 (4.2)
10047065	Vascular disorders	16	5.63 (3.44)	5.6 (60.44)	2.48 (0.95)	5.59 (3.42)
10077536	Product issues	3	12.64 (4.07)	12.62 (32.03)	3.65 (0.74)	12.59 (4.05)

### 3.3 PTs level adverse drug reaction analysis

The top 10 signal strengths of ADRs of amiodarone and dronedarone at the PTs level, ranked by frequency and EBGM, are detailed in Table 5, where we compared them with the adverse reactions spelled out in the drug instructions, using * to mark those not mentioned in the instructions. The top 40 signal strengths of ADRs also presented in Supplementary Table S3.

**TABLE 5:** Top 10 signal strength on the PT level.

PT	SOC	Freq	EBGM (EBGM05)
Amiodarone (sorted by frequency)			
Drug interaction	General disorders and administration site conditions	856	14.24 (13.27)
Hyperthyroidism	Metabolism and nutrition disorders	758	143.63 (132.67)
Dyspnoea	Respiratory, thoracic and mediastinal disorders	607	2.98 (2.75)
Electrocardiogram QT prolonged	Investigations	592	20.23 (18.6)
Toxicity to various agents	Injury, poisoning and procedural complications	465	5.6 (5.1)
Bradycardia	Cardiac disorders	432	21.06 (19.11)
Pulmonary toxicity	Respiratory, thoracic and mediastinal disorders	424	156.16 (140.58)
Interstitial lung disease	Immune system disorders	375	22.39 (20.17)
Hypothyroidism	Metabolism and nutrition disorders	346	33.33 (29.89)
Atrial fibrillation	Cardiac disorders	345	9.97 (8.95)
Dronedarone (sorted by frequency)			
Atrial fibrillation	Cardiac disorders	371	40.09 (35.89)
Dyspnoea	Respiratory, thoracic and mediastinal disorders	204	3.75 (3.25)
Blood creatinine increased	Investigations	123	21 (17.51)
Cardiac failure	Cardiac disorders	116	14.45 (11.99)
Heart rate decreased	Investigations	66	19.69 (15.41)
Drug interaction	General disorders and administration site conditions	62	3.86 (3)
Palpitations	Cardiac disorders	53	4.59 (3.49)
Heart rate increased	Investigations	51	5.76 (4.37)
Dysphagia^a^	Gastrointestinal disorders	47	4.8 (3.6)
Arrhythmia	Cardiac disorders	44	9.36 (6.94)
Amiodarone (sorted by EBGM)			
Myxoedema coma	Metabolism and nutrition disorders	122	597.66 (457.42)
Electrocardiogram T wave alternans^a^	Investigations	18	480.93 (231.27)
Mitochondrial aspartate aminotransferase increased	Investigations	8	858.28 (182.23)
Thyrotoxic crisis	Psychiatric disorders	100	224.92 (180.24)
Iodine overload	Metabolism and nutrition disorders	5	487.66 (148.8)
Pulmonary toxicity	Respiratory, thoracic and mediastinal disorders	424	156.16 (140.58)
Accessory cardiac pathway^a^	Cardiac disorders	6	378.65 (140)
Hyperthyroidism	Metabolism and nutrition disorders	758	143.63 (132.67)
Thyroiditis^a^	Endocrine disorders	178	147.69 (125.91)
Electrocardiogram RR interval prolonged	Investigations	8	276.86 (123.81)
Dronedarone (sorted by EBGM)			
Atrial fibrillation	Cardiac disorders	371	40.09 (35.89)
Cardiac death	General disorders and administration site conditions	10	65.68 (35.11)
Cardiac ablation^a^	Surgical and medical procedures	11	57.85 (31.86)
Cardioversion^a^	Surgical and medical procedures	7	47.85 (22.69)
Pulmonary toxicity	Respiratory, thoracic and mediastinal disorders	23	31.68 (20.98)
Atrial flutter	Cardiac disorders	21	30.12 (19.57)
Blood creatinine increased	Investigations	123	21 (17.51)
Pulmonary fibrosis	Respiratory, thoracic and mediastinal disorders	35	22.3 (15.96)
Heart rate decreased	Investigations	66	19.69 (15.41)
Glomerular filtration rate decreased	Investigations	24	20.79 (13.89)

^a^ The instruction does not mention.

The top 3 frequent adverse safety signals for amiodarone were drug interaction (n = 856), hyperthyroidism (n = 758), and dyspnoea (n = 607); the top 3 largest EBGM05 values were myxoedema coma (EBGM05 = 457.42), electrocardiogram T wave alternans (EBGM05 = 231.27), and mitochondrial aspartate aminotransferase increased (EBGM05 = 182.23). The adverse signals not mentioned in the instructions, for example, were electrocardiogram T wave alternans (n = 16, EBGM05 = 231.27), accessory cardiac pathway (n = 11, EBGM05 = 140), and thyroiditis (n = 178, EBGM05 = 125.91). The top 3 frequent adverse safety signals for dronedarone were atrial fibrillation (n = 371), dyspnoea (n = 204), and blood creatinine increased (n = 123); the top 3 largest EBGM05 values were atrial fibrillation (EBGM05 = 35.89), cardiac death (EBGM05 = 35.11), and cardiac ablation (EBGM05 = 31.86). The adverse signals not mentioned in the instructions, for example, were cardiac ablation (n = 11, EBGM05 = 31.86), cardioversion (n = 7, EBGM05 = 22.69), and dysphagia (n = 47, EBGM05 = 3.6). The whole findings are presented in Supplementary Table S1 completely, where 277 newly identified ADRs of amiodarone and 41 newly identified ADRs of dronedarone were marked using *. The analysis of the real-world study based on the FAERS database also provides great reference value for the revision of the instructions for amiodarone and dronedarone.

### 3.4 Comparison of main organ toxicity signals

**FIGURE 1:**
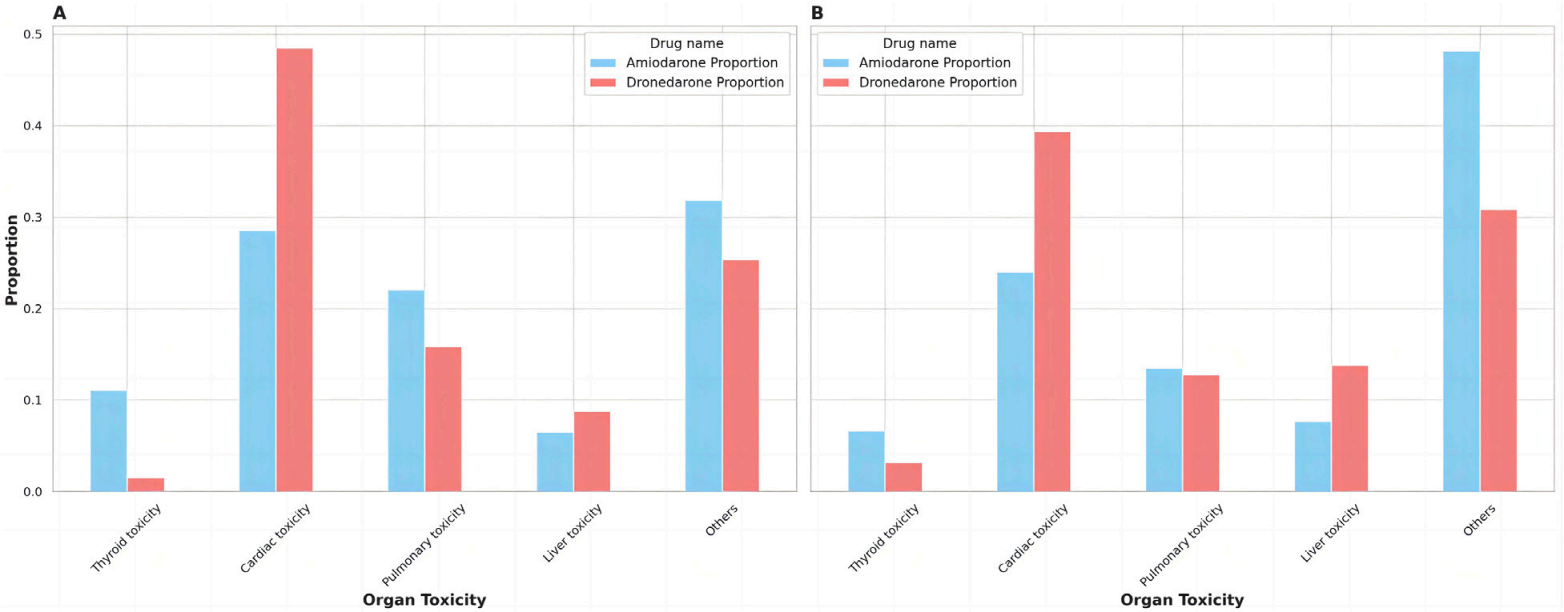
The distribution of organ toxicity for amiodarone and dronedarone. **(A)** Proportion of ADRs; **(B)** Proportion of ADR Types.

The main adverse effects of amiodarone are concentrated in cardiac toxicity, pulmonary toxicity, thyroid toxicity, and liver toxicity, while dronedarone was designed to address the limitations of amiodarone. Our deeper comparison assessed their main adverse reactions in Table 6, allowing us to directly compare the strength of the adverse reaction signals. The organ toxicity distributions of the number of ADRs and types of ADRs at the PTs level are shown in Figure 1, and then displayed and compared in Table 7.

**TABLE 6:** Major signals of thyroid toxicity, cardiac toxicity, pulmonary toxicity and liver toxicity sorted by EBGM.

Drug	PT (Top 3)	Freq	ROR (lower_95% CI)	PRR (χ2)	IC (IC025)	EBGM (EBGM05)
Thyroid toxicity						
Amiodarone	Myxoedema coma	122	1366.01 (1045.48)	1348.1 (72739.98)	9.22 (8.48)	597.66 (457.42)
	Thyrotoxic crisis	100	287.39 (230.31)	284.31 (22313.85)	7.81 (7.09)	224.92 (180.24)
	Hyperthyroidism	5	180.29 (166.54)	165.67 (1,07,520)	7.17 (6.9)	143.63 (132.67)
Dronedarone	Hyperthyroidism	24	17.24 (11.52)	17.08 (361.94)	4.09 (2.81)	17.01 (11.37)
	Thyroid function test abnormal	4	10.71 (4.01)	10.7 (35.08)	3.42 (0.76)	10.67 (4)
	Blood thyroid stimulating hormone increased	5	6.23 (2.59)	6.22 (21.89)	2.64 (0.18)	6.21 (2.58)
Cardiac toxicity						
Amiodarone	Electrocardiogram T wave alternans	13	872.09 (419.37)	870.88 (6231.96)	8.91 (6.82)	480.93 (231.27)
	Accessory cardiac pathway	6	585.02 (216.31)	584.64 (2262.04)	8.56 (5.74)	378.65 (140)
	Electrocardiogram RR interval prolonged	8	373.14 (166.86)	372.82 (2201.00)	8.11 (5.73)	276.86 (123.81)
Dronedarone	Atrial fibrillation	371	47.42 (42.44)	40.49 (14197.96)	5.33 (4.96)	40.09 (35.89)
	Cardiac death	10	67.02 (35.83)	66.75 (637.13)	6.04 (4.15)	65.68 (35.11)
	Cardiac ablation	11	58.94 (32.46)	58.69 (614.78)	5.85 (4.04)	57.85 (31.86)
Pulmonary toxicity						
Amiodarone	Pulmonary toxicity	424	191.27 (172.19)	182.59 (65445.62)	7.29 (6.94)	156.16 (140.58)
	Tracheal compression	4	428.92 (134.5)	428.74 (1219.26)	8.26 (5.05)	306.53 (96.12)
	Pulmonary fibrosis	258	47.06 (41.47)	45.78 (10844.01)	5.46 (5.04)	43.94 (38.73)
Dronedarone	Pulmonary toxicity	23	32.22 (21.33)	31.93 (683.84)	4.99 (3.68)	31.68 (20.98)
	Pulmonary fibrosis	35	22.72 (16.26)	22.42 (712.61)	4.48 (3.4)	22.3 (15.96)
	Organising pneumonia	8	16.31 (8.13)	16.26 (114.12)	4.02 (1.96)	16.2 (8.08)
Liver toxicity						
Amiodarone	Hepatitis acute	28	13.34 (9.19)	13.3 (314.76)	3.72 (2.52)	13.15 (9.06)
	Drug-induced liver injury	133	10.31 (8.68)	10.18 (1091.49)	3.33 (2.76)	10.09 (8.49)
	Mixed liver injury	13	13.9 (8.04)	13.88 (153.39)	3.78 (2.09)	13.71 (7.93)
Dronedarone	Hepatitis acute	7	12.37 (5.88)	12.33 (72.7)	3.62 (1.46)	12.3 (5.85)
	Hepatic failure	18	6.54 (4.11)	6.5 (83.79)	2.7 (1.25)	6.49 (4.08)
	Hepatic necrosis	3	11.98 (3.85)	11.96 (30.06)	3.58 (0.66)	11.93 (3.84)

**TABLE 7:** The distribution of organ toxicity for amiodarone or dronedarone.

Organ toxicity	Type	Amiodarone (n, %)	Dronedarone (n, %)	Z-Statistic	Z *p*-value	Fisher odds ratio	Fisher *p*-value
	Total	**467 (100.00)**	**94 (100.00)**				
Thyroid toxicity	ADR types	31 (6.64)	3 (3.19)	1.28	0.201	8	0.244
Cardiac toxicity	ADR types	112 (23.98)	37 (39.36)	−3.08	0.002	0.42	0.003
Pulmonary toxicity	ADR types	63 (13.49)	12 (12.77)	0.19	0.851	1.5	1.000
Liver toxicity	ADR types	36 (7.71)	13 (13.83)	−1.92	0.055	0.72	0.070
Others	ADR types	225 (48.18)	29 (30.85)	3.08	0.002	1.38	0.002
	Total	**17,471 (100.00)**	**2,153 (100.00)**				
Thyroid toxicity	ADRs	1,935 (11.08)	33 (1.53)	13.91	<0.001	2.16	<0.001
Cardiac toxicity	ADRs	4,991 (28.57)	1,044 (48.49)	−18.9	<0.001	0.49	<0.001
Pulmonary toxicity	ADRs	3,849 (22.03)	341 (15.84)	6.62	<0.001	1.07	<0.001
Liver toxicity	ADRs	1,130 (6.47)	189 (8.78)	−4.04	<0.001	0.52	0.0001
Others	ADRs	5,566 (31.86)	546 (25.36)	6.14	<0.001	2.08	<0.001

First “Total”: Represents the number of unique adverse reaction (ADR) types for amiodarone or dronedarone. Second “Total”: reflects the total count of all recorded ADRs for amiodarone or dronedarone.

The top 3 ADRs of thyroid toxicity, cardiac toxicity, pulmonary toxicity, and liver toxicity sorted by EBGM for both amiodarone and dronedarone are shown in Table 6. We could still find thyroid toxicity for dronedarone use, such as hyperthyroidism (n = 24, EBGM05 = 11.37), thyroid function test abnormal (n = 4, EBGM05 = 4) and blood thyroid stimulating hormone increased (n = 4, EBGM05 = 4). The pulmonary toxicity of amiodarone and dronedarone was similar, both have pulmonary toxicity (n = 424, EBGM05 = 140.58 vs. n = 23, EBGM05 = 20.98) and pulmonary fibrosis (n = 258, EBGM05 = 38.73 vs. n = 35, EBGM05 = 15.96). Hepatitis acute (n = 28, EBGM05 = 9.06 vs. n = 7, EBGM05 = 5.85) of liver toxicity was also listed for amiodarone and dronedarone.

There were 467 kinds of ADR types for amiodarone and 94 kinds of ADR types for dronedarone, with 17,471 ADRs for amiodarone and 2,153 ADRs for dronedarone found in the study. We sorted them into different organ toxicity groups in Supplementary Table S2, presented them as Figure 1, and analyzed them in Table 7. Amiodarone had a higher proportion of ADR types compared to dronedarone in thyroid toxicity, pulmonary toxicity, and liver toxicity, but the differences were not significant. Conversely, dronedarone had a significantly higher proportion of ADR types compared to amiodarone in cardiac toxicity (39.36% vs. 23.98%, *p* ˂ 0.05). Moreover, significant differences in the proportion of ADRs were observed between the two drugs in all categories. Amiodarone had a significantly higher proportion of ADRs compared to dronedarone in thyroid toxicity (11.08% vs. 1.53%, *p* ˂ 0.001) and pulmonary toxicity (22.03% vs. 15.84%, *p* ˂ 0.001), while dronedarone had a significantly higher proportion of ADRs compared to amiodarone in cardiac toxicity (48.49% vs. 28.57%, *p* ˂ 0.001) and liver toxicity (8.78% vs. 6.47%, *p* ˂ 0.001). These differences highlight the varying organ-specific toxicities of the two drugs, which can inform treatment choices and toxicity management strategies.

## 4 Discussion

Based on data from the FAERS database from 2011 Q3 to 2023 Q3, the study used ROR and PRR as the primary assays. Then, we used the BCPNN algorithm to construct a joint probability model between topotecan and the AEs identified by ROR and PRR. Next, we applied the EBGM algorithm to convert the risk association into corresponding risk indices and screened for significant high-risk combinations of the drugs and ADRs identified by the above three algorithms. Finally, this study provides a reference for safe and reasonable clinical use of the drugs.

The study analyzed a total of 9,295 reports of ADRs attributed to amiodarone use and 2,485 reports of ADRs attributed to dronedarone use, mainly originating from the United States (44.29% vs. 73.20%). The relatively late initial approval (July, 2009) in the United States may explain why dronedarone is mainly reported in the United States. Both drugs were more frequently reported among the elderly, aligning with the demographic typically at higher risk for atrial fibrillation (Joglar et al., 2023). It’s imperative to highlight that age-related information was absent for nearly a quarter of the patients (21.89% vs. 32.43%), potentially skewing the results. Notably, the proportion of male reports is significantly higher than that of female reports for amiodarone, which is in line with our daily medical practices, while more reports of females than males were found for dronedarone. The restriction of dronedarone use in severe patients may be the explanation (Schweizer et al., 2011). In addition, hospitalization was the most frequently reported serious outcome among both drugs (49.14% vs. 23.30%), but a high proportion of unknown outcomes for dronedarone may have a significant impact on actual outcome analysis.

### 4.1 System organ classes level analysis

In the disproportionality analysis of SOC levels, amiodarone focused on Cardiac disorders, respiratory disorders, metabolism disorders (mainly thyroid disorders), investigations and general disorders, which was in agreement with the CAST (Cardiac Arrhythmia Suppression Trial) study (Echt et al., 1991), where the common ADRs in patients were thyroid dysfunction, hepatic dysfunction, and pulmonary toxicity, rather than cardiac disorders as the most commonly found ADRs. Dronedarone also focused on cardiac disorders, investigations, and respiratory disorders, and this was not in high agreement with the clinical trials (Hohnloser et al., 2009; Køber et al., 2008; Connolly et al., 2011), where the severity and frequency of ADRs varied instead of concentrating on certain organs or systems. In the SOC level analysis, cardiac disorders were somewhat biased because the applicable disorders were also grouped into PTs level.

### 4.2 New adverse reaction signals

After obtaining the results of all PT level ADR signals for amiodarone and dronedarone, the signals were ranked according to their frequency and EBGM. The higher the frequency, the more valuable the excavation, and the higher the EBGM (EBGM05), the higher the risk combinations of the drugs and ADRs were found. After comparing the drug instructions separately, it was found that both showed new ADR signals that were not mentioned in the instructions.

There were 277 ADRs of amiodarone and 41 ADRs of dronedarone not mentioned in the instructions. The top 3 ADR signals not mentioned in the amiodarone label were electrocardiogram T wave alternans (n = 16, EBGM05 = 231.27), accessory cardiac pathway (n = 11, EBGM05 = 140), and thyroiditis (n = 178, EBGM05 = 125.91). Electrocardiogram T wave alternans and accessory cardiac pathways were both additions to the diversity of results in cardiac medical examinations. Thyroiditis is a kind of thyroid toxicity of amiodarone that has been widely reported for years but not officially included in the drug instructions (Ylli et al., 2021). SIADH was identified in association with amiodarone (n = 19, EBGM05 = 3.2), although the signal strength was low, SIADH may be related to amiodarone’s effects on hypothalamic ADH release, possibly due to neurotoxicity or direct renal impact (Hannon and Thompson, 2010). The top 3 ADR signals not mentioned in the dronedarone instructions were cardiac ablation (n = 11, EBGM05 = 31.86), cardioversion (n = 7, EBGM05 = 22.69), and saliva altered (n = 3, EBGM05 = 12.69). The cardiac ablation and the cardioversion both suggested that the use of dronedarone had proarrhythmic side effects that may need medical intervention or surgical procedures (Rizkallah et al., 2016). While saliva altered was not directly highlighted as a common issue with dronedarone, any changes in taste or mouth discomfort could potentially impact saliva production and consistency since some patients report changes in taste, such as a metallic taste or loss of taste, which can indirectly affect saliva production. T-wave alternans could be found in both amiodarone and dronedarone, it may caused by electrolyte imbalances, particularly hypokalemia and hypomagnesemia, which disrupt the cardiac action potential and increase the susceptibility to arrhythmias. Additionally, its proarrhythmic effects can exacerbate the heterogeneity of repolarization across the myocardium, contributing to the occurrence of T-wave alternans (Narayan, 2006; Roden, 2008).

### 4.3 Comparison of organ toxicity

A deeper analysis included a summary of all organ toxicity signals for both amiodarone and dronedarone through statistical analysis of the ADR profiles, including the severity of ADRs, the number of ADRs, and the ADR type. According to Figure 1, it can be seen that cardiac toxicity accounted for the largest parts of both, which makes sense as all antiarrhythmic drugs tend to have proarrhythmic side effects at the same time. It should be noted that dronedarone had a higher risk of severe cardiac toxicity events (cardiac death, EBGM05 = 35.11) compared with amiodarone (cardiac arrest, EBGM05 = 6.32), which explains why it is restricted to people who have risk factors for cardiovascular events (Joglar et al., 2023). Moreover, dronedarone showed higher proportions of cardiac toxicity in both ADRs (48.49% vs. 28.57%, *p* ˂ 0.001) and ADR types (39.36% vs. 23.98%, *p* ˂ 0.05). Meanwhile, dronedarone reduced the types, numbers, and severity of thyroid toxicity. Unlike amiodarone, which has various types and numbers of thyroid toxic ADRs and even severe ADRs such as myxoedema coma and thyrotoxic crisis, this is consistent with previous research (Hohnloser et al., 2009). Moreover, the liver toxicity of ADRs distribution is higher for dronedarone compared to amiodarone (8.78% vs. 6.47%, *p* ˂ 0.001), as evidenced by two reported cases of severe liver injury requiring organ transplantation, which prompted the European Medicines Agency to perform a comprehensive review of all available data on potential liver toxicity caused by dronedarone (European Medicines Agency, 1998). Post-marketing data, however, found an association between the use of Class III antiarrhythmics and the onset of acute liver injury, mainly driven by amiodarone (Grimaldi-Bensouda et al., 2018). Pulmonary toxicity of ADRs distribution, however, is higher for amiodarone compared to dronedarone (22.03% vs. 15.84%, *p* ˂ 0.001), though more and more dronedarone pulmonary toxicity events are being reported (Hernández Voth et al., 2012; Stack et al., 2015; Chen et al., 2024).

### 4.4 Limitations

Several limitations of this real-world observational study with large-sample data must be acknowledged. Firstly, the voluntary nature of reporting to the FAERS database makes it vulnerable to underreporting, potentially leading to incomplete data. This might result in less severe or common adverse events being underrepresented, while more serious or rare events could be disproportionately reported, introducing a risk of data omission or information bias (Yang et al., 2023; Zhao et al., 2023; Du et al., 2024c). This selective reporting can lead to an overrepresentation of severe adverse events and an underrepresentation of less severe ones. Secondly, explicit causality isn’t mandatorily required for data submission, meaning the reports can highlight safety signal strengths without necessarily indicating actual risk levels. Thirdly, the absence of comprehensive data on the total number of patients treated with amiodarone or dronedarone precludes accurate calculation of ADRs’ true incidence from FAERS data. In signal detection, a strong signal might result from high reporting rates rather than a high true incidence, potentially leading to misinterpretation of the drug’s safety profile. Additionally, the clinical interpretation of the ADR signals, particularly for cardiac ablation and cardioversion, may be confounded by the drug’s lower efficacy, which necessitates more aggressive treatments rather than reflecting direct toxicities. This highlights the importance of cautious clinical interpretation and the need for future research to further explore the relationship between drug efficacy and ADR reporting. Finally, our analysis predominantly focused on exploring the correlation between amiodarone or dronedarone and ADRs, designating the drug’s role as a “preferred suspect.” This approach implies a focus on the direct correlation between the drug and ADRs without delving into the potential effects of multi-factorial confounding factors, such as secondary suspect drugs, concomitant medications, and multi-drug interactions.

## 5 Conclusion

The study applied pharmacovigilance analysis methods to the FAERS database to detect safety signals of ADRs linked to amiodarone and dronedarone treatment and provided some complementary ADR signals that were not mentioned in the instructions. Through further analysis of organ toxicity, it was found that dronedarone had a higher proportion of ADR types and ADRs for cardiac toxicity but a lower proportion of ADR types and ADRs for thyroid toxicity compared to amiodarone. Given these findings, clinicians should implement tailored monitoring strategies, focusing on thyroid and pulmonary health for amiodarone users and cardiac toxicity for those on dronedarone. Long-term follow-up and regular assessments are crucial to detect delayed adverse events. Additionally, informed discussions with patients about the specific risks of each drug are essential for shared decision-making. It’s imperative to closely monitor for new and unforeseen ADRs upon administering the medication, as certain life-threatening adverse events necessitate prompt detection and intervention. Overall, this study provides a reference for the reasonable and safe clinical use of amiodarone and dronedarone.

## Data Availability

Publicly available datasets were analyzed in this study. This data can be found here: https://fis.fda.gov/extensions/FPD-QDE-FAERS/FPD-QDE-FAERS.html.
