# Supplementary material for: Adverse drug reaction signals mining comparison of amiodarone and dronedarone: a pharmacovigilance study based on FAERS
Source: Front Pharmacol. 2024 Oct 22;15:1438292. doi: 10.3389/fphar.2024.1438292 (PMC11534617; doi:10.3389/fphar.2024.1438292)
Supplement: Supplementary file 2 [file Table3.pdf]

TABLE S3. Top 40 signal strength on the PT level sorted by EBGM (*: The instruction does not mention).

Amiodarone			Dronedarone		
**PT**	**Freq**	**EBGM （EBGM05）**	**EBGM （EBGM05）**	**Freq**	**PT**
myxoedema coma	122	597.66 (457.42)	40.09 (35.89)	371	atrial fibrillation
electrocardiogram T wave alternans*	18	419.81 (232.13)	65.68 (35.11)	10	cardiac death
mitochondrial aspartate aminotransferase increased	8	858.28 (182.23)	57.85 (31.86)	11	cardiac ablation*
thyrotoxic crisis	100	224.92 (180.24)	47.85 (22.69)	7	cardioversion*
iodine overload	5	487.66 (148.8)	31.68 (20.98)	23	pulmonary toxicity
pulmonary toxicity	424	156.16 (140.58)	30.12 (19.57)	21	atrial flutter
accessory cardiac pathway*	6	378.65 (140)	21 (17.51)	123	blood creatinine increased

hyperthyroidism	758	143.63 (132.67)	22.3 (15.96)	35	pulmonary fibrosis
thyroiditis*	178	147.69 (125.91)	19.69 (15.41)	66	heart rate decreased
electrocardiogram RR interval prolonged	8	276.86 (123.81)	20.79 (13.89)	24	glomerular filtration rate decreased
corneal deposits	39	161.55 (114.85)	34.97 (13.06)	4	sinus arrhythmia
cornea verticillata*	22	180.17 (113.9)	39.6 (12.69)	3	saliva altered*
secondary hyperthyroidism	10	223.51 (111.34)	14.45 (11.99)	116	cardiac failure
device pacing issue	11	210.74 (108.97)	17.01 (11.37)	24	hyperthyroidism*
toxic nodular goitre	8	238.41 (108.63)	19.46 (11.02)	12	extrasystoles*
lymphoid tissue hyperplasia	21	163.26 (102.55)	18.13 (10.9)	15	ventricular extrasystoles*
urine iodine increased*	5	282.33 (101.67)	14.92 (10.4)	30	heart rate irregular
tracheal compression*	4	306.53 (96.12)	17.95 (8.96)	8	atrioventricular block first degree*

myxoedema	11	171.03 (89.74)	14.95 (8.47)	12	torsade de pointes*
arteriosclerotic gangrene*	3	357.62 (89.42)	16.2 (8.08)	8	organising pneumonia*
keratopathy*	31	127.92 (87.86)	11.61 (7.2)	17	ventricular tachycardia*
toxic goitre	5	233.23 (86.57)	9.36 (6.94)	44	arrhythmia
protein bound iodine increased*	3	804.63 (83.69)	13.57 (6.77)	8	cardiac pacemaker insertion*
goitre congenital*	5	223.51 (83.44)	11.14 (6.31)	12	blood urea increased
thyroiditis acute*	9	150.87 (74.54)	18.87 (6.07)	3	atrial tachycardia
thyroxine free increased	23	113.19 (73.44)	18.72 (6.02)	3	bradyarrhythmia
fasting*	5	191.58 (72.82)	12.3 (5.85)	7	hepatitis acute
BRASH syndrome*	43	93.77 (68.53)	14.93 (5.59)	4	orthopnoea*
tachycardia induced cardiomyopathy*	9	137.94 (68.49)	10.9 (5.19)	7	cardiac flutter
